# Raritas: a program for counting high diversity categorical data with highly unequal abundances

**DOI:** 10.7717/peerj.5453

**Published:** 2018-10-09

**Authors:** David B. Lazarus, Johan Renaudie, Dorina Lenz, Patrick Diver, Jens Klump

**Affiliations:** 1 Museum für Naturkunde, Berlin, Germany; 2 Leibniz-Institut für Zoo- und Wildtierforschung, Berlin, Germany; 3 Divdat Consulting, Wesley, AR, USA; 4 CSIRO, Mineral Resources, Kensington, NSW, Australia

**Keywords:** Software, Point-counting, Rarity, Data standards, Micropaleontology, Biostratigraphy, Biodiversity, Ecology, Python, Range chart

## Abstract

Acquiring data on the occurrences of many types of difficult to identify objects are often still made by human observation, for example, in biodiversity and paleontologic research. Existing computer counting programs used to record such data have various limitations, including inflexibility and cost. We describe a new open-source program for this purpose—Raritas. Raritas is written in Python and can be run as a standalone app for recent versions of either MacOS or Windows, or from the command line as easily customized source code. The program explicitly supports a rare category count mode which makes it easier to collect quantitative data on rare categories, for example, rare species which are important in biodiversity surveys. Lastly, we describe the file format used by Raritas and propose it as a standard for storing geologic biodiversity data. ‘Stratigraphic occurrence data’ file format combines extensive sample metadata and a flexible structure for recording occurrence data of species or other categories in a series of samples.

## Introduction

### Human observations as a source of scientific data

Quantitative data about many aspects of the natural world are collected in modern science with the use of instruments, but a substantial amount of observational data is still collected by human observation. This is particularly common in ecology, organismal biology and behavioural sciences, where numeric data on the frequencies of occurrences of biologic phenomena are desired, but the objects or phenomena to be counted are too complex to identify by instruments or fully computerized image analysis systems. Up until the widespread use of desktop computers, such counts were done mostly either with the aid of mechanical counters (including arrays of several buttons, to allow counting of multiple categories) or tallied by hand on printed list forms. Both methods are slow and require re-entering the count values into a computer afterwards before analysis, adding additional time and possibilities for error. Computer ‘point-counting’ programs can in principle replace these methods and at the same time provide additional functions that mechanical methods cannot, such as continuous statistical summaries of the data as it is being collected, which provides useful feedback to the observer on how complete or accurate the dataset being collected is.

Despite these obvious advantages, counting programs have yet to fully replace manual methods. There are many reasons for this including cost, inflexibility, compatibility and inadequate ease of use. Numerous inexpensive or free simple tally counter programs are available that can replace mechanical counter buttons, for example, dozens of simple smartphone/tablet apps, or more sophisticated desktop apps, for example, Versacount: (Kim & DeRisi, 2010) (Table 1). None of these, however, are well suited to counting larger numbers of categories, which is common in ecology, and in related fields such as paleontology. The need to count many objects in many categories is particularly acute in biodiversity related disciplines, for example, field surveys of species diversity, or species counts of fossil assemblages in micropaleontology. In such studies the diversity of objects and total numbers of objects available for study are both very high. Several programs (Table 1) have been developed to assist in biodiversity assessments (e.g. ‘OrgaCount’: http://www.aquaecology.de/; ‘Beecam’: http://www.advansee.com/bee_home_en). As many micropaleontologists work in commercial (oil industry) settings, there are also several sophisticated counting programs available (many as commercial products) for counting large numbers of microfossils: Polpal (Nalepka & Walanus, 2003); Foramsampler (Mcgann et al., 2006); Counter (Zippi, 2007); Stratabug (Stratadata, 2014); Bugwin (Bugware, 2016) (Table 1). These programs, whether for biologists or industrial micropaleontologists, are excellent choices for those specific groups of researchers and the research (e.g. biostratigraphy, standardized ecologic surveys) for which they were developed. These programs include many specialized features such as stratified ecologic sampling, cell volume calculations, biostratigraphic event recording and range charting, or petrologic thin section analyses. The large number of additional features however also have a price, as these programs tend to lack flexibility, adaptability and/or ease of use outside the specialized domain for which they were developed. Many are also closed-source, expensive, platform specific, and are dependent on the commercial provider to maintain. There is thus a need for a program that is relatively simple; and less specialized, thus adaptable to counting a variety of different types of objects. It should also be free and open source and work with different operating systems. Most importantly, it must be as easy to use as mechanical methods, since a program that is significantly slower will, based on our experience, normally be rejected by users. Users often need to count thousands of objects (see ‘rarity’ below), and an even marginally slower data entry method will create an unacceptable cumulative loss of the user’s time. This is particularly true in counting objects such as microfossils, or in field biodiversity surveys, where vast numbers of specimens are available and can be quickly identified by the user, making data entry the time-limiting factor in data collection.

**Table 1:** Table of other biodiversity counting programs.

Name	Extent and intended purpose	Entry method	Metadata	Rare taxa support	Free test	Platforms	Support	Cost	Usage
Bugwin	Includes biostratigraphy plotting features and table output; mini-taxa catalogue with images	GUI button entries; adding from non-hierarchical master list via pause to create new button	Moderate; fixed	No rare category mode	Free trial no longer active	Windows only	Actively updated	Single licence $200/800 (university/industry)	Closed
Counter	Counting only	GUI buttons or flat list chooser tho content is same	Very limited (comments)	No; tho (by external tracking of scan effort/common abundances can retrospectively multiply counts above choosable cut-off)	Free trial	Win/Mac	Last software update 2011 (webpage content 2008)	Single licence $150	Closed
Orgacount	Part of large plankton ecology/sql db package. Very complex manual (>100 pages). includes, for example, cell size/volume; thumbnail images of taxa; several tabular output formats (‘reports’)	GUI button or keyboard. Complex nested list-like web GUI for metadata	Extensive, many fixed but customization via unlimited optional fields; linked to taxon db	Yes: ‘freeze count’ for a taxon and enter counted area. Also can use multiple taxa lists (tho must pause to swap lists)	Free trial of basic version after registration at website	Browser-based so not OS dependent	Active, though most activity dates between 2011–2016	Free ‘basic’ to 2,000 Euro ‘professional.’ Customization costs extra.	Closed
Polpal	Embedded in general plotting/analysis package for palynology	Keyboard-codes data entry	Unknown	No rare category mode?	No free version (that includes counter)	Windows only	Last update 2012	Single licence Euro 307.50	Closed
Stratabugs	Part of large sql database-centred oil industry well management/analysis system	GUI button entry	Metadata-substantial/in database	Yes—set cut-off for common vs rare taxa	Yes, on approval	Windows only	Actively updated	Academic-varies by case; 5–12K£ business (plus annual support fees)	Closed
Versacount	Counting only	Keyboard single keys only (GUI buttons only for delete)	None	No rare count mode	Free software	Windows, tho uses cross-platform language and GUI library	Last update 2010	Free	Download at sourceforge, 3.2K lines Perl and tK

**Notes:**

Representative counting programs for biodiversity research. Information is that accessible online, via inquiry to provider or test of program, if free trial available.

### Rarity

**Figure 1:**
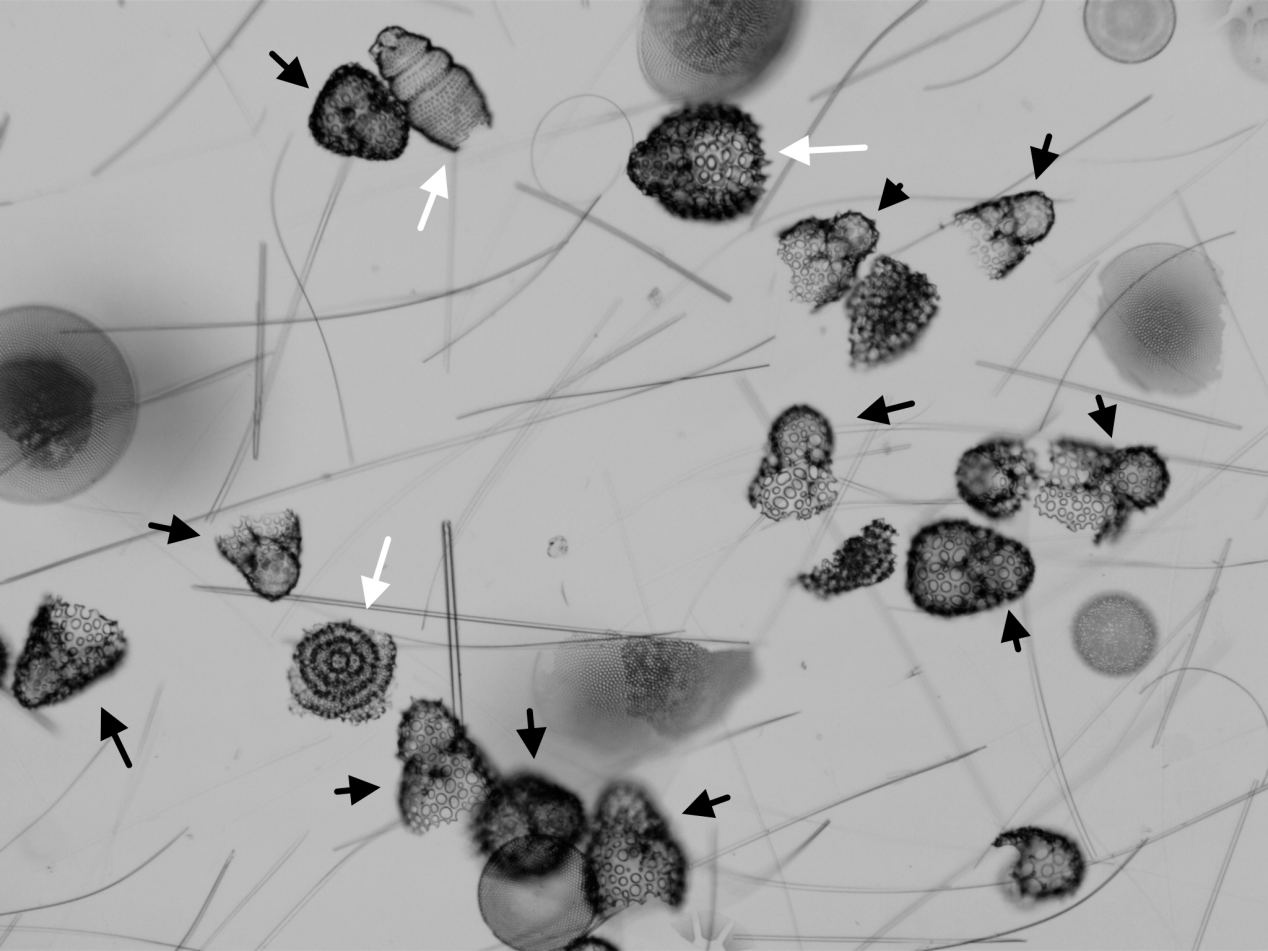
Assemblages with common and rare taxa. Microfossil assemblage as seen in the microscope (late Pleistocene, Southern Ocean, ODP Site 751). Specimens marked by black arrows all belong to *Antarctissa strelkovi* or *A. denticulata*. Other radiolarian species are marked by white arrows. Unmarked individuals are not targets for counting—broken radiolarians and diatom valves. Most individuals in this target assemblage belong to just a few species (particularly *A. strelkovi* and *A. denticulata*), making discovery of rarer taxa difficult.

**Figure 2:**
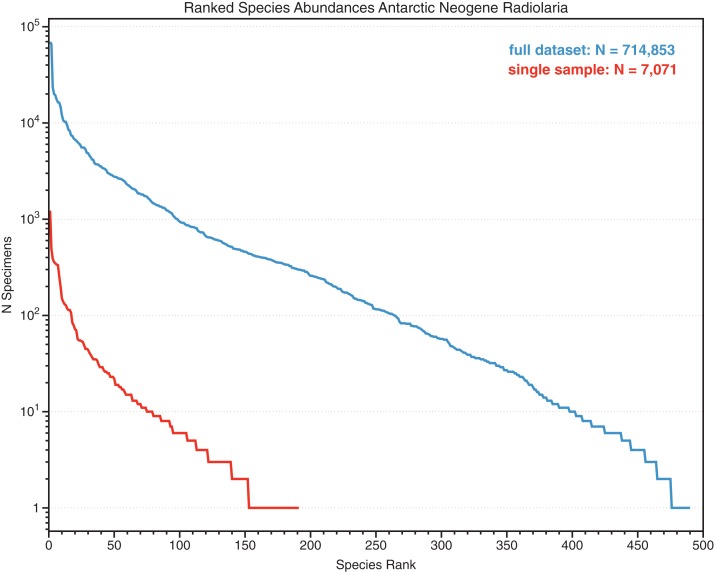
Ranked relative abundances of fossil radiolarian species in single samples and combined multi-sample datasets. Counts of species, sorted by abundance, of Neogene Southern Ocean radiolarian assemblages, showing total dataset (several dozen samples) and a single sample (Deep-sea drilling sample ODP 751A-6H-6, 98–100 cm). Despite a total count of 7,071 specimens within the single sample, the majority of the species are represented by six or fewer individuals. From data in Renaudie & Lazarus (2013) SOM.

**Figure 3:**
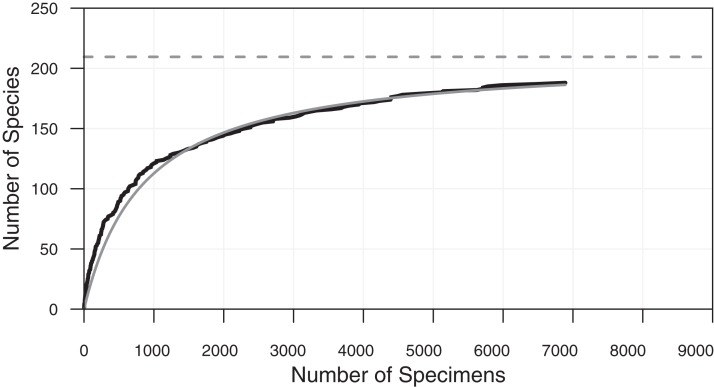
Cumulative diversity vs sample size curve and estimated true diversity for a single sample. Species-accumulation curve on a typical sample (sample ODP 751A-6H-6, 98–100 cm shown in Fig. 1). Bold black curve is the species-accumulation curve; light grey curve is a de Caprariis type curve-fit; dashed light grey line its asymptote (i.e. species diversity at infinite sample size). From Renaudie & Lazarus (2013).

In addition to the general need for flexible, efficient counting programs, there is also a specific need to count objects which have very different relative abundances. Many classes of objects in the observable world show a characteristic pattern of unequal relative abundances that can be approximated by power laws, including incomes, internet traffic, plankton sizes and the sizes of interstellar mineral grains (Mathis, Rumpl & Nordsieck, 1977; Reed & Hughes, 2002; Buonassissi & Dierssen, 2010). Biologic entities, in particular species abundances in ecology and paleontology, also typically show such distributions, with a few species being relatively common, and the remainder uncommon or quite rare (Preston, 1948; Brown et al., 2002). The degree to which objects are unequally common in samples is measured by indices such as evenness (Hill, 1973). Counting objects at random from such unevenly distributed populations results in many counts of the few common species, but very few counts of rarer species. For example, in both the complete dataset, and in individual samples, counts of fossil radiolarians in Neogene Southern Ocean sediments show a few very common species, and many rare species (Renaudie & Lazarus, 2013; Figs. 1 and 2). Even with >700,000 individuals, a substantial fraction of the species are represented by 10 or fewer individuals. Thus, in order to encounter at least one individual of all rare species very large numbers of specimens need to be examined. For example, several thousand individuals needed to be examined in order to recover 95% of the estimated total species diversity (ca 200 species) in the single sample counted in Figs. 2 and 3.

Ecologists and paleontologists thus sometimes decide to base studies only on the small number of species that are relatively common and thus whose abundances are easy to quantify. Many applied micropaleontologic studies, for example, use the environmental preferences of a relatively small number of common species to reconstruct past environmental conditions (Imbrie & Kipp, 1971; CLIMAP Project Members, 1976). Not all scientific questions can however be addressed by examination of only a small number of common species. Each biologic species, whether common or rare, is unique: all species have the potential to contribute to ecosystem function and, over the longer term, to evolutionary change. Biodiversity research in particular is concerned about documenting total species richness and understanding threats to it, for example, how current and past environmental change affects it. The findings of such research feed into important decisions on biodiversity conservation, land use and other global issues (i.e. the ‘Rio’ Convention on Biological Diversity: www.cbd.int). Reasonably accurate estimates of total diversity—crucial in biodiversity studies—can only be made when the majority of the diversity has been counted. Extrapolations from less complete data tend to have unacceptably high error values (Colwell et al., 2012). There is thus a major effort to understand the total species richness of modern and past biologic systems (Mora et al., 2011) and, consequently, the need to collect quantitative data on many rare species (Roberts et al., 2016).

One approach to achieving this is based on the human ability to scan large populations to identify a subset of target individuals much more rapidly than the same person could fully identify and record the identity of each individual in the population. As an example, it is much faster to scan a large crowd of people to identify a single category of persons of interest (‘tall men with beards’), than to identify each person in a crowd and record all of their names. Similarly, one can quickly skip individuals belonging to a specific category to target other individuals (e.g. skip all men). Biologists and paleontologists collecting data on rare species make use of this ability by first counting all individuals encountered to identify common species, then, mentally blocking out the common species, continuing to count only species that are not in the ‘common’ group. In this ‘rare category’ mode, individuals of common species can be scanned over much more rapidly, and their counts estimated afterwards based on their abundances in ‘all species’ mode. Larger total numbers of individuals are thereby examined, and a better estimate of total species richness can be obtained (Gannon, 1971; Hinds, 1999; Stevenson, Pan & Van Dam, 2010). A good counting program for such work should offer options that support this style of efficient counting of only rare taxa. This ability is however, to our knowledge, normally not offered in currently available counting programs, which are mostly designed to support counts of smaller numbers of species and individuals in support of applied (paleo) environmental research. The only applications that to our knowledge provide even partial support for this are Orgacount and Stratabugs. The former allows the user to stop counting individual categories and enter the area counted for that category and also the consequent adjustment factor for combining the count with the general counts. Both these must be tracked and calculated for each individual taxon, external to the program, by the user. Stratabugs provides only the ability to stop counting at a limit for some taxa and to continue to count ‘out of count’ (in the program’s terminology) for rare species. It does not provide tracking or entry of the area (effort) expended for these different types of count data and thus the ability to combine the counts into a unified dataset.

Lastly, to our knowledge no existing program provides dynamic feedback on the degree to which the count data reflects the ‘coverage’ (adequacy of sampling) of diversity (*sensu* Colwell et al., 2012), which is important information for maximizing the efficiency of diversity counting for individual samples.

This paper describes a new program—Raritas—which addresses the issues outlined above. Raritas is distinguished from existing programs in several ways: by being simple to use, focussed only on counting, explicitly supporting rare taxa; and by being cross-platform, free and open-source.

## Materials and Methods

Raritas offers a flexible mouse-driven interface for counting highly diverse lists of taxa, including both buttons for more common taxa, and hierarchical menus to select rare taxa. An additional, feature of the program is the definition of a new file format for storing such count data that uniquely combines the data and detailed metadata in a user-friendly spreadsheet style layout. Compiled apps, source code, user guides, sample configuration and output files are all publicly available at https://github.com/plannapus/Raritas.

The program provides explicit support of dual-mode counting (all vs rare only), and indeed this feature is the basis for the program name. In normal mode, all individuals seen are counted. In ‘rare only’ mode, commonly occurring objects are no longer counted: only rare objects are. Not having to pause to enter a count for the most frequently seen object types makes counting rare object categories much faster. However, in order to be able to combine counts for common and rare types together, it is also necessary to know the magnitude of observational effort made in each counting mode, as the total frequencies of common objects are estimated for the ‘rare objects only’ interval based on their frequency in ‘all object’ counting, and the observational effort spent in ‘rare’ mode. A computer program that supports rare-only counting must therefore be able to monitor observational effort in parallel to recording individual object counts. This is provided for by a separate counter for observational effort, a ‘track’ counter which the user updates periodically while counting.

The program’s ease of use involves both ease of configuration as well as ease of use during primary operation. Raritas is configured almost entirely from the contents of a simple tabular type file which can be created easily by users using a spreadsheet program. The file contains a list of the objects (e.g. species) that are to be counted, and how these are to be presented to the user (button labels and other details). This also simplifies the program as there is no need to write code for configuration, other than reading the configuration file.

Detailed metadata are captured for each dataset and saved with the data in the output files. This is often a weakness in other (e.g. commercial) programs where relatively little information is captured. Reliance on program-external metadata capture such as embedding all metadata in filenames is obviously limited in extent, not well structured and in our experience has not been very reliable, particularly when metadata needs to be understandable over the long-term (i.e. by other than the file creators). This file format stratigraphic occurrence data (SOD) format is particularly optimized for paleontology, where biodiversity data is combined with geologic data, but can in principle be adapted to any type of biodiversity data, especially for data for the same set of categories but taken in a series of related samples.

Raritas has been programmed in Python (Van Rossum & Drake, 2010) because it is a popular, well supported, and relatively easy to learn multi-paradigm scripting computer language. Raritas consists of ca. 650 lines of Python code. It runs quickly on all hardware tested (desktop and laptop computers with Intel ‘i’ series processors, running Windows 7–10; OS X 10.9-12). The use of Python, plus the small size of the code, makes customization of the Raritas’s features possible by technically knowledgable users, without the need to employ a professional programmer. Python also provides excellent packages for some functions such as plotting data that allow the program to produce high quality outputs for the user without having to write additional code (e.g. matplotlib). Python is not without problems—installing the various software modules (packages), including packages used by other packages (dependencies) that an application needs can be difficult for a non-specialist, and depends in part on the local python environment used. Raritas is therefore offered both as a fully bundled program (double-clickable) with all needed packages included for MacOS X 10.11+, as well as for Windows 7 and 10; and also as source code. The bundled program provides ease-of-use for non-specialists; the source code version customizability.

In addition to Raritas, we mention here, but do not discuss further in this paper, an older prototype version of the program: RaritasVox. This initial version of the program was used to collect a large amount of data in mouse counting mode, but the voice module was never developed or tested beyond the prototype stage. RaritasVox was programmed in Java in order to make use of specialized libraries for voice recognition, and to insure speed, which is needed for the complex task of voice recognition. RaritasVox provides most, though not all of the features of the main Python version in mouse-based counting. In addition, it provides a unique option to register counts directly from voice input by the user, who simply speaks the category names. Other than features specific to voice entry, the same type of output, setup and configuration files are used as in Raritas. RaritasVox is a relatively complex program with nearly 4,000 lines of Java, and is difficult to properly install due to the required voice recognition libraries. For this reason, and as the voice system was never really used, the VOX version was abandoned and replaced by the newer, simpler, more accessible Python mouse-only version. RaritasVox can however still be downloaded from the public GitHub repository either as a bundled app (a .jar file) or as source code. The bundled version is ca. 100 Mb in size.

### Installation

No special installation procedure is needed for the Raritas program when used as the bundled app. Using the source code version of Raritas (python) requires installing only two python packages (and their dependencies): matplotlib and wxPython (Hunter, 2007; Dunn, 2014). These must be installed using the appropriate python or OS package manager for the user’s python system, which will automatically install any dependencies. Some python distributions already include both packages as part of their standard installation, thus requiring no special installations by the user. Additional details are provided in Appendix S1.

### Configuration file and starting the program

**Figure 4:**
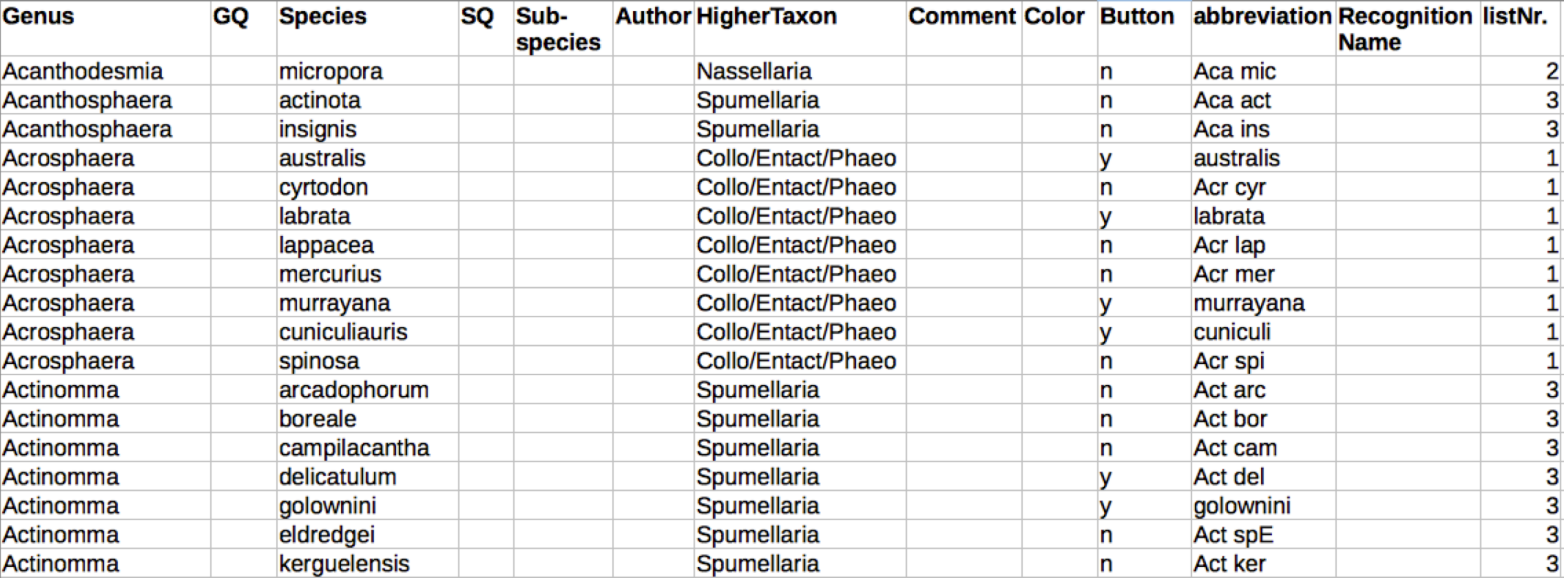
Configuration file to populate interface with category names. Configuration file format (a plain text file, here formatted for easier reading). Only a few fields—‘genus’ and ‘species’ components of a taxonomic name, button (yes/no) are mandatory. A couple fields, for example, ‘recognition name’ are used only by RaritasVox.

Raritas reads a single configuration file on starting—by default, the one previously used, or a new one chosen by the user. The file (Fig. 4; Appendix S2) is in tab-text format and is just a list of taxa names and how each should be presented to the user in the GUI interface. All names are available in the program interface in drop-down list by default. Names can also be shown as buttons (with abbreviations to insure the button label fits). If a second set of names of higher level categories are provided for the primary names, the name list is parsed into multiple lists,, each with drop-down menus, thus providing structure to longer name lists and more rapid access to taxa names.

The Bundled version is started by the usual double-click of the app icon or other standard GUI methods. The source code version of Raritas is started by a standard ‘python raritas.py’ statement (optionally including a path name, if appropriate) at the command line. Once the program starts all interaction takes place via the GUI interface that then appears.

### GUI interface for manual counting

The main elements of the GUI interface are: the metadata window, the counting window, the rare count configuration window and the collector curve window.

### Metadata window

**Figure 5:**
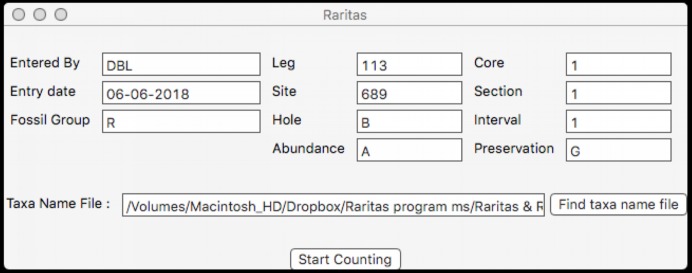
Dialog to enter general sample metadata. Metadata window used for Raritas. Information about the sample to be counted is entered here, including observer, date, class of objects being counted (‘fossil group’), and sample identification information. RaritasVox has additional options (not shown), for example, ‘save list of counted species with diversity’ which, if checked, creates a second output file that gives the entire history of counting.

When the program is first started a window (Fig. 5) appears which provides a pop-up list of primary counting style options (file types), based on the program’s SOD file specification (described below). The next window collects the metadata appropriate for the file type, for example, field names that are used in the rest of the program for the material to be counted. At the moment the program supports two types of primary data, both for microfossil occurrences: assemblages of microfossils from deep-sea sediments obtained by the international deep-sea drilling programs (DSDP, ODP, IODP), or fossils from samples obtained from geologic sections on land, but other types can be defined, including non-paleontologic ones. The metadata window also provides a few run-time options for configuring the interface and behaviour during counting. Importantly, the user chooses which taxa name list configuration file they want to use via a normal file open dialog at this time. When ready the ‘start counting’ button is clicked and the counting window appears.

### Counting window

**Figure 6:**
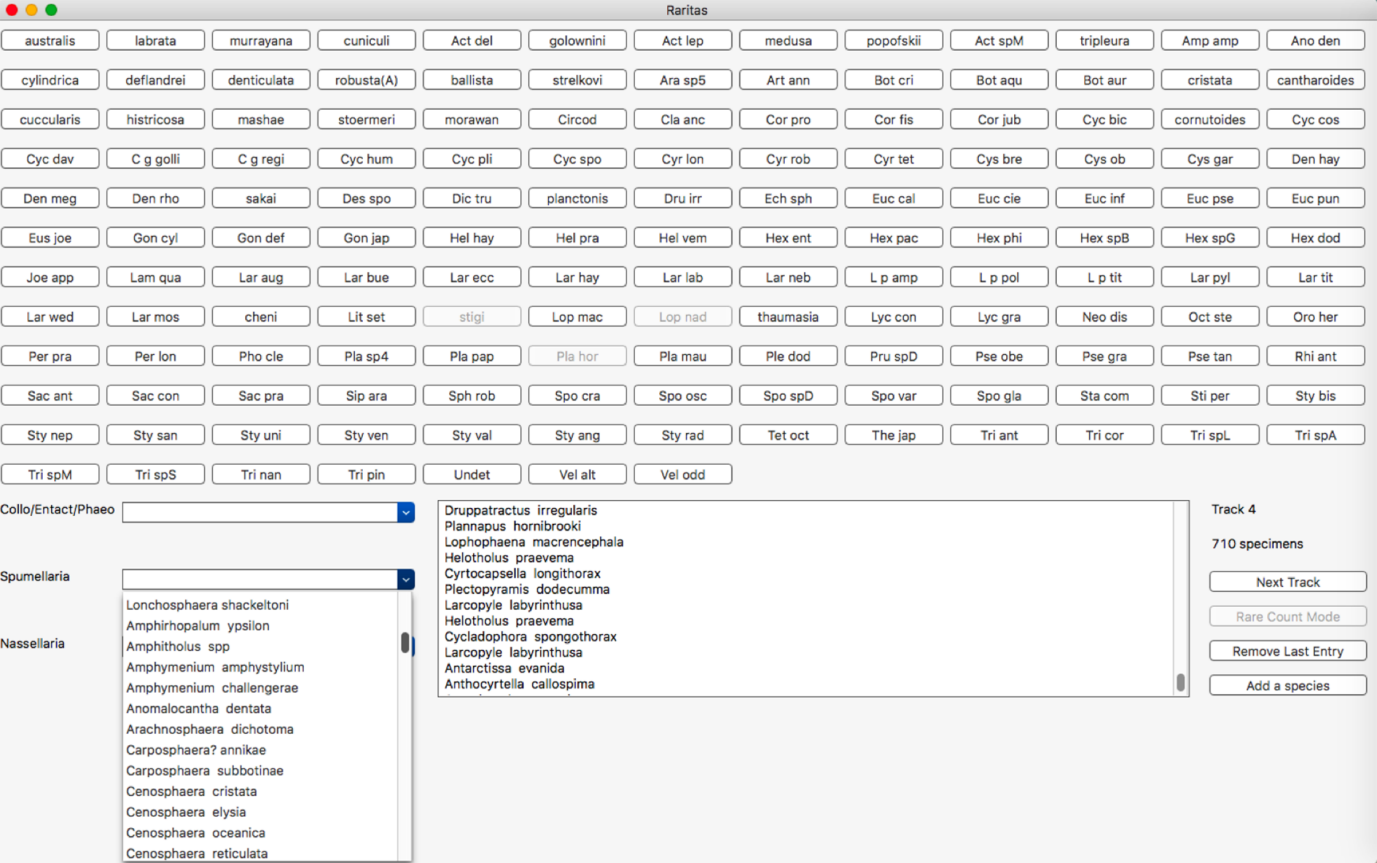
Main counting window with buttons, hierarchical category menus and count status information. Main counting window. Objects to be counted are presented in two forms: an array of clickable buttons in the upper part of the window, and as a set of pop-up lists in the lower left and centre part of the window. The number of lists and their contents is automatically built from the configuration file higher category labels for object entries. Button labels are also taken from this file on start-up. Other buttons or menu items control program behaviour and call up other features, for example, voice recognition (RaritasVox only), show count plot, switch to rare count mode, etc. A scrolling list of the most recently counted objects is shown in the lower middle. The ‘track’ counter and clickable (large rectangular) button are on the lower right and are used to record observation effort in both regular and rare count modes. Note, in this image rare count mode has already been activated; thus, some buttons are greyed out.

The counting window (Fig. 6) is the main window that is used for most interaction with the program. The upper part of the window is populated with the buttons and is meant for counting common species, with labels as defined in the configuration file. Less common taxa are typically shown only in the form of popup lists, organized into higher level categories, again as defined in the configuration file. Putting less common taxa into lists and common taxa on buttons allows most counts to be done quickly with a button, while the comparatively slow process of selecting from a list is reduced to a minimum. Lists are needed however as they can be of arbitrary length, while the number of buttons is limited by screen size. Counting is active whenever the window is present. Clicking on a button or selecting a taxa from the lists adds the species to the count data structures. A list of recently counted objects is given in the sub-window (lower middle of main window). A button is provided to remove the last entry, in case it was erroneously entered. A button on the right counts observational effort (‘Track,’ for number of ‘tracks’ scanned on a microscope slide) and a counter shows the total tracks counted. There is also a button to add a species to a counting list ‘on-the-fly,’ that is, when counting has already begun.

**Figure 7:**
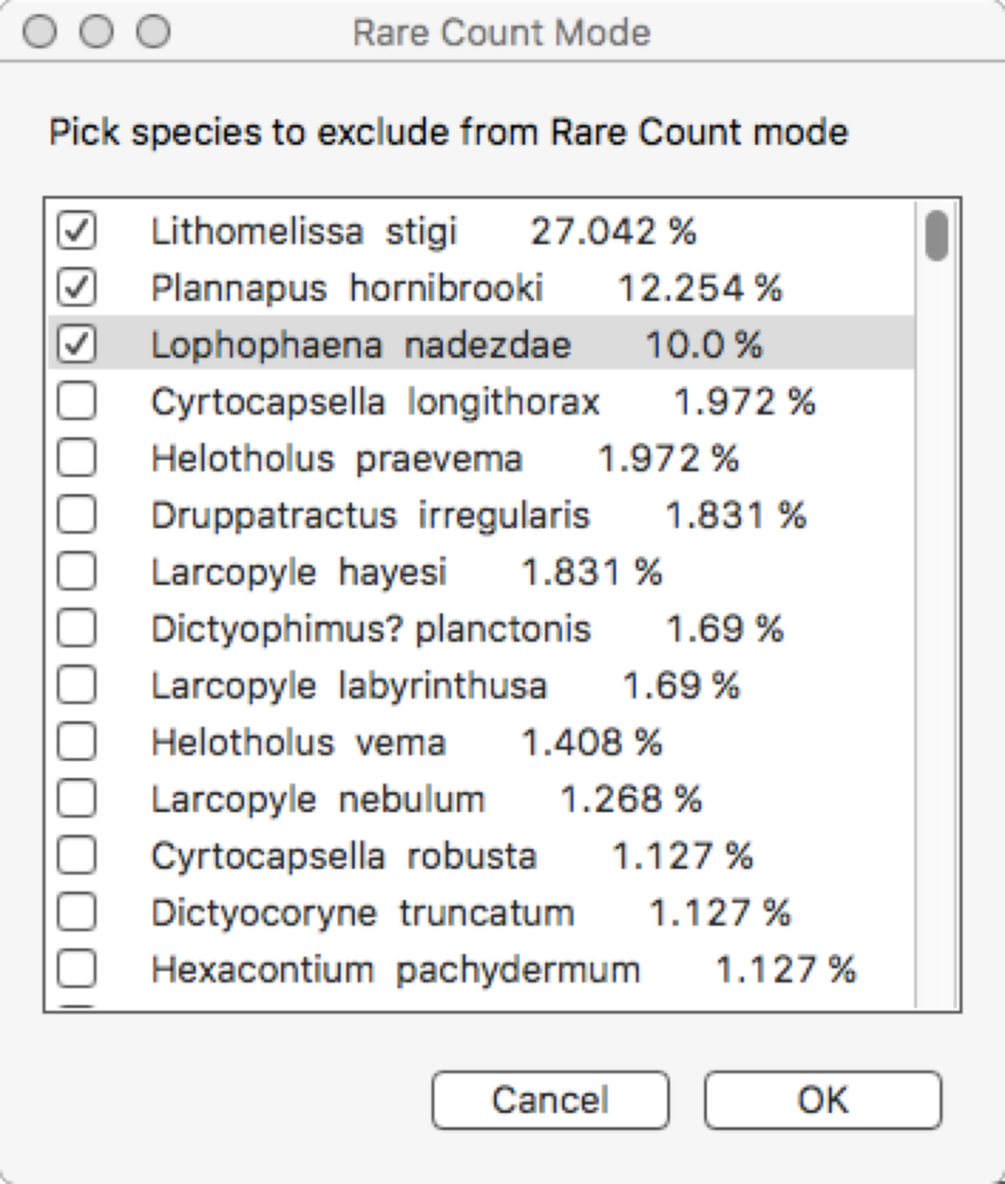
Dialog to configure rare count mode. Configure rare count mode dialog. The object counts list, sorted by count frequencies, is presented and the user selects those objects (here, species names) that will in skipped and no longer counted in rare count mode.

**Rare count mode.** This is the most distinctive feature of Raritas. Clicking on ‘rare count mode’ brings up a dialog box (Fig. 7), where the counted objects are listed in order of descending abundance, and the user can choose which to exclude from further counting. When the dialog is dismissed counting resumes, with, for those taxa to be excluded, the taxa buttons greyed out and pop-up list items inactivated.

**Figure 8:**
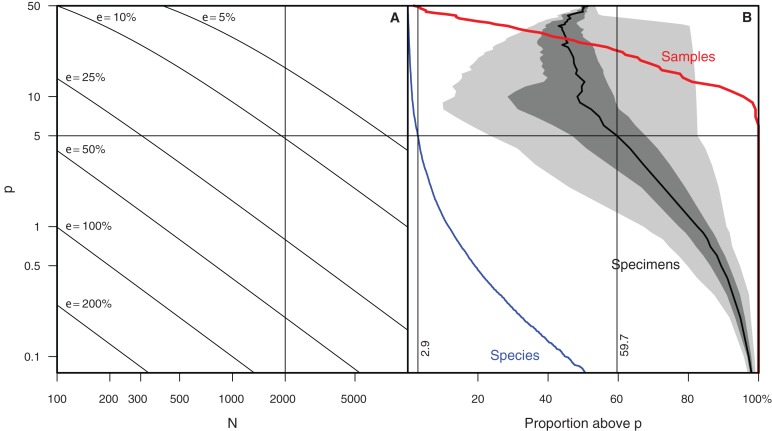
Relationships between sample size and uncertainty of abundance estimates in generalized and actual biodiversity data. (A) Epsilon (size of confidence interval, relative to the abundance value, for a given species relative abundance in a population) plotted on a *p* (percent) vs *N* (number of specimen) landscape. Rule of thumb used in (12) marked by dashed lines (Renaudie & Lazarus, 2013) highlighted. (B) shows, for data reported in Renaudie & Lazarus (2013), red line: the percent of samples that have at least one species with percent higher than *p*; blue line: the percent of species having a proportion higher than *p* in at least one sample, and black line with shading: the cumulative proportion of specimens of species with proportion higher than *p* (mean, inner-quartile range and total range over all 107 samples).

Determining which species to exclude in rare count mode is not trivial. As this is a key feature of Raritas we include the following suggestions, which are based on our experience of counting ca. 700,000 total specimens (several thousand specimens per sample in over 100 samples) for the study published in Renaudie & Lazarus (2013). The number of specimens count value at which the switch is made to rare-only counting, and the percentage threshold for species to be ignored during ‘rare’ count mode should both be chosen to maximize the number of specimens to ignore while minimizing the error in estimating the abundant species percentages. In our own work (Renaudie & Lazarus, 2013), we chose to stop the full count mode when 1,000–2,000 specimens were already counted and to ignore in ‘rare’ count mode species with a percentage higher than approximately 5% of the community. Doing so allowed us to keep the error to ca. 10% of the investigated value. In other words, for a species that was present at 5% abundance in full count mode, the theoretical standard error is slightly below 10% of this 5% value, that is, a theoretical percentage for the species between ca. 4.5% and 5.5%; (Drooger, in Zachariasse et al. (1978)) (Fig. 8). These cut-off values eliminated in our samples 59.7% of the specimens during rare-only mode (median of all samples counted, but varying from one sample to the other). An additional, important criterion that was taken into consideration is that all samples encountered had at least one species above the ‘ignore in rare-only mode’ percent threshold. Using an higher threshold than 5% would have meant that some samples would have had to be counted entirely in full count mode, as no species would have been abundant enough to exclude. In our study, there were on average ca. 3% (mean = 2.9%) of the species above the cut-off threshold per sample (blue and red lines of Fig. 7B).

**Figure 9:**
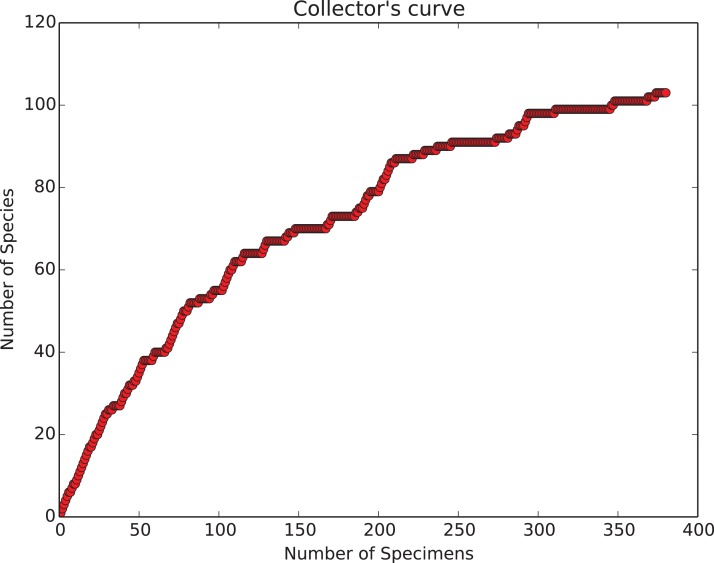
Collecting curve, showing history of cumulative diversity vs sample size. Count plot window, showing a simple graphic of how total diversity of objects (‘species’) is increasing with increased numbers of counted objects (‘specimens’). The window appears whenever the user clicks the ‘show count plot’ button in the main counting window. This graphic is calculated and plotted anew with each invocation. The shape of the curve provides important feedback for the user, see text for details.

**Collection curve.** The ‘show collector’s curve’ menu item brings up the fourth main GUI element—a diversity accumulation plot (Fig. 9) showing the relationship to cumulative total number of object types seen (species) vs total number of objects counted (specimens). For typical biologic data these curves show a roughly logarithmic shape—at first rising rapidly, then, as increasingly species already seen previously are re-encountered, flattening out. The curve’s slope will eventually become zero when all object types in the sample have been detected (compare to Fig. 2). The user can decide when the curve has become close enough to this state for his/her purposes, and thus stop counting only when the data completeness quality is adequate. If a series of samples are counted to the point where they have the same apparent slope at the end of this dynamically generated diversity accumulation curve, they will share the property of being ‘fairly’ sampled, and relative differences in diversity will be shown without bias (Alroy, 2010; Colwell et al., 2012). This type of feedback is important to insuring good quality observations as differing degrees of evenness between samples means that unless sampling is adjusted to achieve the same degree of completeness the observed relative diversities will be biased by differences in evenness (Alroy, 2010; Colwell et al., 2012). Dynamic feedback on sampling adequacy is something that cannot be provided by simple mechanical count systems. It is however rarely implemented in programs known to us.

**Menu commands.** Raritas includes a small number of additional features selected using standard GUI menus. These include options to look at the current counts for all taxa and, importantly for counting large numbers of specimens, the ability to save a partial count for a sample and to reload the partial count data at a later time, resuming counting preserving the cumulative count history and counting mode. Partial count files are in a temporary file format meant only for Raritas’ internal use and is not in the standard SOD output format as described below.

### Output files

#### SOD file format

In addition to the diversity accumulation plots, which can be saved as graphics as often as desired (the matplotlib library used in Raritas supports various file formats, for example, png, pdf, jpg, tif), the program of course saves the primary count data. This necessitates choosing, or creating a format for the data files, as there is no universal community database which would allow a direct upload solution. Despite a great deal of biostratigraphic or other data in the form of ‘species by samples/observations’ having been generated globally for many decades, no generally accepted or even widely known file format exists for such data. Other fields have developed community data formats for such data matrices, for example, the BIOM format for biological observation matrices (McDonald et al., 2012), as well as standard protocols to exchange information directly between computer systems, for example, Darwin Core (Wieczorek et al., 2012). These formats are however of limited use for paleontologic fossil occurrence matrices since they lack any way to store metadata, either in general or for individual samples, that is related to geologic age (sample position in section, formation name, etc.); and the metadata in general are optimized for biologic, not paleontologic observations. One of the major biologic exchange protocols (ABCD: (Berendsohn, 2007), http://wiki.tdwg.org/ABCD/) does have, via the EFG extension (http://www.geocase.eu/efg) the ability to transmit both biologic and geologic data, but is a communication protocol, not storage format, and the XML definition is not readable by normal users.

Within the field of paleontology, data on occurrences, outside of micropaleontology, are dominated by simple taxa lists for a single locality (one sample). This is exemplified by the main data input formats the most widely used paleontology community database PBDB (Alroy et al., 2001), where data are entered, taxon by taxon, for one sample at a time. Within micropaleontology taxa-by-sample data matrices (often referred to as ‘range charts’) are common, but are published in a wide variety of formats specific to individual publications, without metadata in the files. This is also the file format used by the DSDP, ODP, IODP, which have not generally captured micropaleontology data except in a very limited form on-ship, using database entry forms; or simply by archiving data copied from publications, with only minimal metadata, which is stored separately from the data files. Lastly there are several more comprehensive data file formats that are associated with commercial micropaleontology, that is, the oil industry. These formats include metadata, details of stratigraphy etc., but are not compatible with each other and are mostly meant for internal use in proprietary commercial programs, not for open file exchange. Most also tend to be quite user unfriendly, giving sample and taxa names in separate definition blocks from the actual occurrence data, and use long, non-tabular, list type structures that makes comprehension difficult. There is thus a need for a public (non-proprietary) file format that combines metadata and the taxa-by-occurrences data in a single file, provides for geologic age or section information and is easy for scientists to read and use.

We have therefore introduce a new ‘open file format’ format, which Raritas uses to write count data: Stratigraphic Occurrence Data format, which we abbreviate here simply as SOD format. This format in fact was originally was developed for internal use by us prior to the development of Raritas. The initial goal of SOD format was to merge metadata and occurrence data in a large number of user typed fossil occurrence matrix files. These were being digitized from the literature for upload into a database that provides a micropaleontologic equivalent to the PBDB: NSB (Lazarus, 1994; Spencer-Cervato, 1999). This database reports occurrences of microfossils in deep-sea sediment sections, and the data are mostly derived from studies that report the occurrences in the form of simple samples by species tables, one table per section, per higher fossil group. SOD file format itself is deliberately meant to be visually similar to the source publication data tables, being essentially an enhanced version of the publication’s tabular data matrix. This makes the file easily read by users, and also makes the transcription (keying-in) of data from publications into the file relatively simple. In some cases in fact, where a publication file is available in digital form, all that is needed is reformatting some of the fields, rather than re-entry of primary values. SOD format, however, is significantly different from an ‘ordinary’ user data table in that it is based on a formal, extendable definition of content, particularly metadata fields.

**Figure 10:**
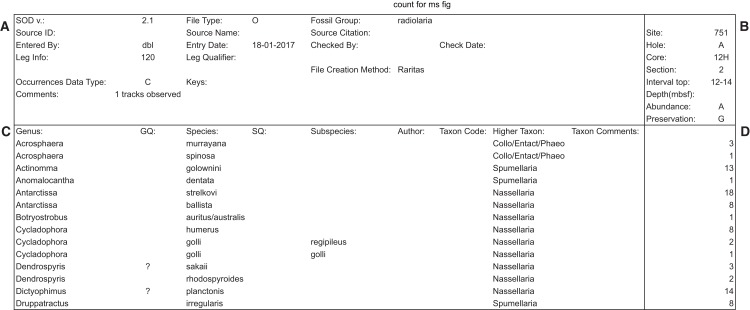
Example of SOD file format with data blocks framed. Example of SOD file output (the main data output file produced by Raritas), with the four main areas (blocks) marked by bold lines. Metadata about the data file is stored (A), object labels and linked data such as author names, if known, are in (C), sample information is in (B), and the actual counting data in (D). In output from the Raritas program only a single column of data is created but the SOD format definition permits the sample name and count values to repeat indefinitely (to the right of this figure). Note that only a few selected rows are shown here—the full file has ca. 400 taxa name.

The file is laid out in four graphical blocks: general metadata—upper left; taxa metadata—lower left; sample metadata—upper right; and the occurrence data—lower right (Fig. 10). Flexibility is provided for in two ways. The individual fields in each block can be populated by different actual data types, depending on the overall record type as determined by the ‘file type’ field. Currently there are only two defined file types, for ‘ocean deep-sea drilling’ data and more traditional ‘land section’ data (O and L, respectively). These differ both in general metadata (Site location vs geographic name and geographic coordinates), and in the way in which sample names are structured: deep-sea drilling samples (‘O’ files) use a consistent Site-Hole-Core-Section-Interval format, while land sections are more variably defined, but usually include some combination of geologic formation, vertical position in section and sample name (usually unique to each study); with additional information often recorded on geologic age or biostratigraphic zone and lithology. SOD ‘L’ formatted files include all these fields. Within the broad constraints on total fields available, the number of file types using this layout is open to indefinite expansion. The SOD layout itself is also extensible, as the version is written in the first metadata field in each file. The field definitions and thus the data expected in each field are determined by these control fields, and different layouts can be defined, for example, with additional rows for sample name fields. This flexibility however requires a separate source of information that defines, for the user and programmer, what the field contents must be for each ‘file type’ or SOD version number. These definition requirements are the fundamental difference between regular data files as found in the literature, and the SOD format. The definitions are given in two ways (which also allows cross checking for data consistency). First, the tabular file definition requires full labelling—each cell, row or column that holds data has an adjacent cell with fixed text content defining the data cell(s) adjacent, so that the content resembles a simple key: value non-relational database structure. This means the files are largely self documenting, and provides sufficient explanatory information to users so that they can create new data files from a template file (containing labels but no data values). Second, programs, such as Raritas, that write and read SOD files are expected to have a definition table of some sort which gives the location and meaning of each cell for each file type and each SOD version. Currently this is implemented in a table in the NSB database and used by programs (both a Python script and an R procedure at present) that read and upload SOD data into the NSB system. In the form of a definition list, the information could also be included (e.g. as a second ‘page’ in a spreadsheet file) with the data files themselves. At present, the structure of defined SOD formats is hard-wired into Raritas, although the format is much more flexible and can accommodate many more types of data than the current version of Raritas itself. Future versions of Raritas ideally should be modified to read the fields needed for the metadata window, and output data file formats, directly from a SOD definition file. A full list of current SOD field definitions and additional details on the format are given in Appendix S3.

Over 500 files have been created in SOD format, both typed or edited by users as described above, or generated by the Raritas program during counting of microfossils. Raritas generates, and reads, only data for one sample at a time, but otherwise the output is identical to that used for complete sample by taxa matrices in other SOD files. SOD formatted files are not intended to replace more complex, formally controlled, computer-to-computer data exchange formats, defined in XML or other systems. SOD is best viewed as complementary, providing a user accessible format that encourages the capture of the metadata needed to adequately document stratigraphic occurrence data, which until now has often not been done.

### Diversity vs number of specimens

The program outputs, in addition to the main count data, the cumulative diversity vs. number of counted objects history as a simple tab-text data file. This data can be useful for fitting rarefaction curves in subsequent data analyses.

## Results

The degree to which biodiversity assessments can be improved using our software depends on a variety of factors—the distribution of taxon abundances (evenness) and absolute diversity of the target population(s) being counted; and on how many specimens are counted before switching to rare count mode. Improvement in diversity assessment depends also on the ability of the user to mentally mask out taxa and focus only on those not excluded. Most people can easily keep a ‘skip’ list of several taxa in mind when counting, but not a much larger list, for example, a dozen or more taxa. Thus the improvement in counting with Raritas tends to be best when the abundances are significantly uneven and the total diversity is less than a few hundred categories. In the example shown in Figs. 1 and 7 of this paper, from Antarctic Pleistocene radiolarian assemblages, by eliminating the six most common species (cumulative abundance of >74% of the specimens in the sample) nearly 3/4 of the specimens can be skipped, allowing an effective sampling size for the rarer taxa that is 4× what would have been possible by counting all specimens. In practice we have found that we more typically increase our effective sample size by 2–3× by using rare count mode. These increased effective sample sizes significantly improve the accuracy of diversity estimates, although the precise amount will depend on total sample size, evenness and absolute diversity (Colwell et al., 2012).

**Figure 11:**
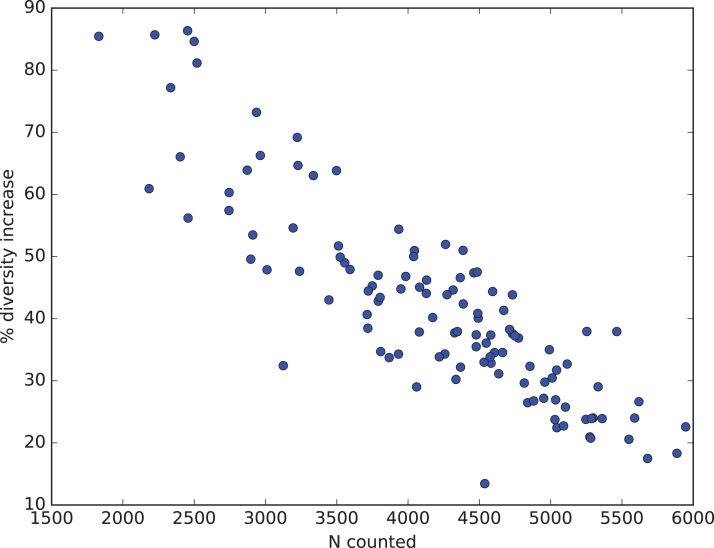
Percent diversity capture improvement from rare mode counting. Relationship in Neogene radiolarian data (Renaudie, 2012; Renaudie & Lazarus, 2013; Renaudie, this paper: full data and script in SOM Appendix S4) between number of specimens actually counted (*x*-axis) and percent improvement in total observed diversity (species richness, *y*-axis) by using Raritas and switching to rare count mode when the count total in a sample reached between 1,000 and 2,000 specimens. Improvement values are calculated for each sample by estimating how many species would have been found using the same total count for the sample but not using rare count mode, and are calculated by rarefaction from the observed diversity and count values (values are means based on 1,000 replicates).

How all these factors influence diversity assessment in practice are illustrated from Renaudie’s earlier work on Neogene radiolarians from Southern Ocean deep-sea sediments (Renaudie, 2012; Renaudie & Lazarus, 2013; Renaudie, this paper: SOM Appendix S4). The goal of this research has been to obtain, to the extent resources permit, a complete inventory of radiolarian diversity in our samples. To this end substantially larger numbers of specimens in individual samples (median > 4,000) were counted than is typical for much micropaleontological or other biodiversity research, and rare count mode was engaged only after 1,000–2,000 specimens had been counted in normal mode. This has an effect on the percent improvement in observed diversity, as sample counts before changing to rare count mode had already captured a substantial fraction of the total diversity and the data was thus already showing a noticeable reduction in the improvement (number of new species found) vs numbers of specimens counted (i.e. the curve relating these two variables was already beginning to flatten out: see Fig. 3). Nonetheless the improvement was substantial, with an increase in observed diversity of ca. 40% (Fig. 11). This figure also illustrates that the improvement is indeed strongly sample size dependent. In our data, the smallest count values benefited the most, with observed diversity increasing by >80%. With sample sizes <1,000 specimens, which is typical for most micropaleontologic work, the observed diversity in our material would be more than double that observed without using rare count mode.

## Discussion and Conclusions

The current (initial) version of the program has some limitations, for example, in dealing gracefully with large numbers of buttons (more than a hundred) and/or large numbers of taxa lists, at least in combination with small computer screens. Rare count mode could also be improved. Currently it is set only once, and all taxa to be skipped must be selected at this time. A per-taxon selection option as is offered by Orgacount would for some types of data collection be an improvement. Despite these limitations, the program described here provides a useful tool for counting populations with large numbers of categories and unequal abundances of individuals in categories. Raritas, as programmed, is best suited to micropaleontology studies, but with only minor modification can be adapted to many other uses in biodiversity research and other fields. The SOD definition provides a flexible, internally documented yet easy to read file format for storing and exchanging occurrence data, either for individual populations or matrices with multiple sets of observations. The Raritas program has proved itself in actual use over several years in the junior author’s research group in Berlin. As noted above, it has been used to count >700,000 specimens belonging to several hundred different species in >100 radiolarian microfossil assemblages, as part of a study of biodiversity change in the Southern Ocean over the last 20 my and has been used by several other individuals, including students, in other micropaleontology projects, on a variety of computers.

## Supplemental Information

10.7717/peerj.5453/supp-1 Supplemental Information 1 User guide, sample files and SOD definition. Archive (zip format) with detailed user guide, installation tips, example configuration and output files, and detailed definition of currently defined versions of the SOD format.

## Additional Information and Declarations

### Data Availability

The following information was supplied regarding data availability: GitHub: https://github.com/plannapus/Raritas.
