# Supplementary material for: Raritas: a program for counting high diversity categorical data with highly unequal abundances
Source: PeerJ. 2018 Oct 9;6:e5453. doi: 10.7717/peerj.5453 (PMC6183511; doi:10.7717/peerj.5453)
Supplement: Supplemental Information 1 — Archive (zip format) with detailed user guide, installation tips, example configuration and output files, and detailed definition of currently defined versions of the SOD format. [file peerj-06-5453-s001.zip › Appendix 1 - User Guides/User Guide for Raritas app.pdf]

User Guide for Raritas.app

A companion document to the publication:

This is for the bundled, no-voice python app only. Please see separate guide for the VOX version, and comments on how to install and use the python source code version of Raritas in the publication. Instructions here are based on the Mac OS version but should be virtually the same for the Windows version.

Software version :	0.6
Manual version :	1.0
Date :	June 12, 2018
Manual written by	Dave Lazarus

1 Installation and initial set-up

1.1 Installation

Program installation is not required; simply unpacking the zip archive should create a folder with the app in a ready to use state. The app can be run from any folder, though for convenience it is best to have it in the same folder as the configuration file described in the next paragraph.

1.2 Configuration

Support file configuration is needed before using the program. Raritas' main counting window is populated with clickable labeled buttons and drop-down menus of count object names that come from a configuration file. This is a simple tab-text file that can be created with and read by any spreadsheet program (e.g. Excel, LibreOffice Calc), and can be given any name desired. The format of the file is a set of 13 columns, with a single header row. These columns match those used in the SOD-OFF data specification as described in the publication, and are specified in detail in Appendix 2. The columns are defined briefly here. Only a few are required and these are noted below. Unneeded fields can be left blank.

Genus	genus name. Required.
GQ	qualifier for the genus name, e.g. '?'
Species	species name. Required.
SQ	qualifier for the species name, e.g. 'cf.'
Subspecies	for a subspecies or variety name, if used
Author	original or modifying author of the name.
HigherTaxon	the distinct values entered in this column will be used to generate the drop-down lists. If left blank for a taxon it will not appear in the list of the higher taxon. Note that this allows the program to be configured so that the lists are either comprehensive or have only those taxa not shown as buttons. It is not necessary to enter any values at all in this column. If no values are given no lists appear and counting is done only with buttons. Lists are displayed in a vertical row in the main counting window. Too many distinct values here will create a long stack of onscreen lists that may not fit onto the computer's screen. There is no limit to the number of items within a single list.
Comment	optional. Does not appear in the program and is meant for use by the person managing the configuration file.
Color	currently not used by Raritas but only by RaritasVOX, but column should be left in file for compatibility.
onButton	Required, allowed values are 'y' or 'n'. If 'y' then a button will appear on the main counting window so one-click counts can be made for this taxon. The upper limit for buttons is not fixed but should be less than ca 200, or locating buttons will become difficult. Large numbers of buttons also require a larger minimum space to display. If there are too many buttons for the available screen size the edge of the window will be offscreen. If this happens reduce the number of buttons or use a larger screen. The order of the buttons in the program is determined by the order given in the configuration file. The first taxon row in the configuration file marked 'y' becomes the uppermost left button in the main counting window, and each additional row in the configuration file marked 'y' appears in normal 'text flow' order in the counting window (left to right-hand edge of the counting window, then a new row beginning on the left below the first). The number of buttons per row in the counting window is calculated internally by the program to balance space and button size. Note that the program expects to find at least one 'y' value, and will not run without at least one button appearing in the counting window.
abbreviation	If the 'onButton' field was set to 'y' then a short text string should be given here which will be used as the button label. Blanks etc are allowed. Again, no fixed limit on size but fewer characters (e.g. 12) usually work better for formatting purposes with larger numbers of buttons (e.g. > 100).
RecognitionName	field used only by RaritasVOX but left in for compatibility.
listNr.	field used only by RaritasVOX but left in for compatibility.

Note that Raritas uses the full taxon name as a way to correctly manage count data. Thus each name (genus+gq+species+sq+subspecies) needs to be unique within the configuration file.

2 Running the program

Start the program by double-clicking it. Due to peculiarities of the underlying python modules used in Raritas trying to (re)start the program using operating system shortcuts (e.g. 'Recent Items' in Mac OS' apple menu) do not always work. Starting the app from its icon is recommended.

You will first enter general information about the counting in two dialogs before the main counting window appears.
The first dialog sets the data type. Currently you can choose 'Deep-Sea Core' for samples from DSDP, ODP or IODP sections; or 'Land Section' for a generic sample name and metadata format.

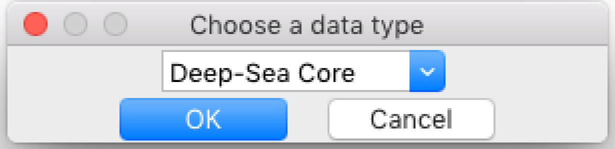

Start-up Screen.

The second dialog collects 1) the sample metadata which will be written out along with the count data in the output file; and 2) the configuration file name (described above) that has the definitions of the taxa (or other objects) to be counted.

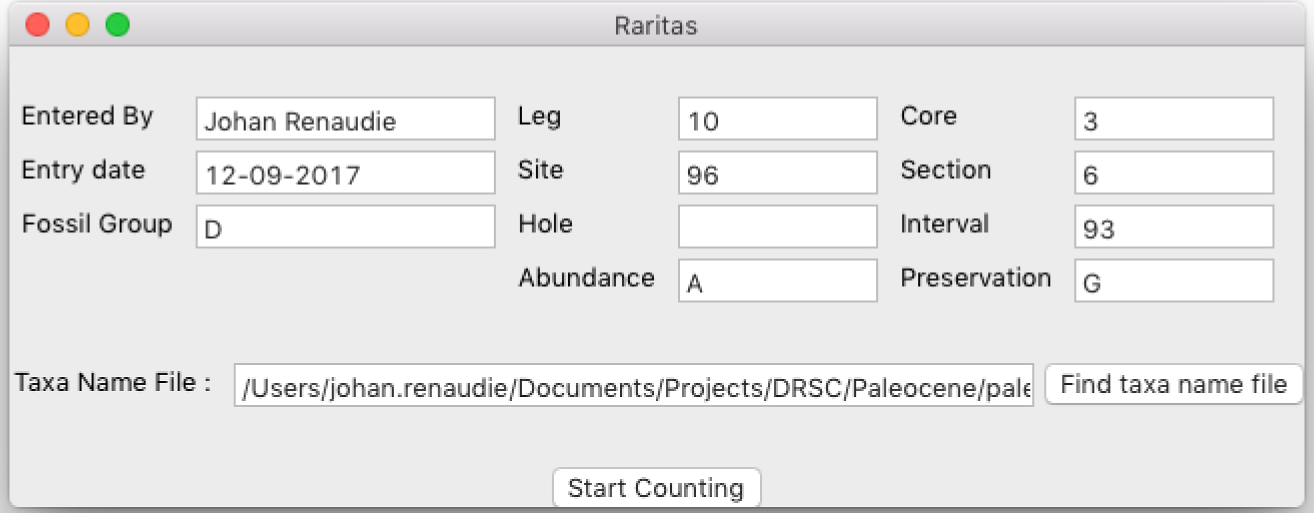

Metadata screen (here for Deep-Sea Drilling samples).

You can enter the filename as text (including the path to the file, if it is not in the same directory as the application), or browse the computer to find the file using the 'Find taxa name file' button. The program remembers the location and name of the configuration file last used and will reuse this automatically if no new configuration file is specified. Currently the sample metadata fileeld names as given below are hard-wired into the program. They can easily be changed in the source code version of the program however. The field contents are not of any special importance to running the program. They are just text values that are written out again to the saved data file, so they can in principle be used to enter any desired structured metadata, such as field locations, trap numbers or

other types of sampling information; or even left blank. The descriptions below are simply to assist users who are entering paleontologic data from geologic samples.

The common metadata fields are:

Entered By	name of person generating the data
Entry Date	date data collected
Fossil Group	name or letter code, e.g. Radiolaria, R, benthics
Abundance	overall abundance of fossils in the sample or slide
Preservation	overall quality of preservation of the fossils

The Deep-Sea Core specific metadata fields corresponds to the standard nomenclature for deep-sea drilling sample names (e.g. 120-751-A-12H-3, 53-57 cm):

Leg	In the above example, 120
Site	In the above example, 751
Hole	In the above example, A
Core	In the above example, 12H
Section	In the above example, 3
Interval	In the above example, 53-57cm

The Land Section specific metadata fields are:

Formation	name of geologic unit the sample comes from
Sample Name	name of section, outcrop, and individual sample
Geographic Name	locality name
Latitude, Longitude	coordinate information, if available
meter level	measured location if sample one of series in a profile/section
Age	name or numeric value if known
Zone	alternate or supplement to Age, if known
Lithology	text description of sediment/rock

Click on the 'start counting' button to show the main counting window and begin counting. The sample metadata dialog remains onscreen but is no longer needed, and can be closed if desired.

3 Main program counting window

This window is divided into 5 function areas - a block of individual taxa counting buttons across the top, drop-down lists of taxa names in the lower left, a list of all specimens counted in a lower middle panel, buttons to control program operation at the lower right, and running counters for specimens and tracks just above the control buttons. The window can be resized. and the individual elements will grow/shrink to the space available. This can be useful. Buttons for example will grow/shrink to the space available, so you can make counting buttons larger to accomodate longer button labels. You can pull the base of the window down if needed to expose all the drop-down lists, if you have a large number of them.

3.1 'Normal' (or 'all taxa') counting

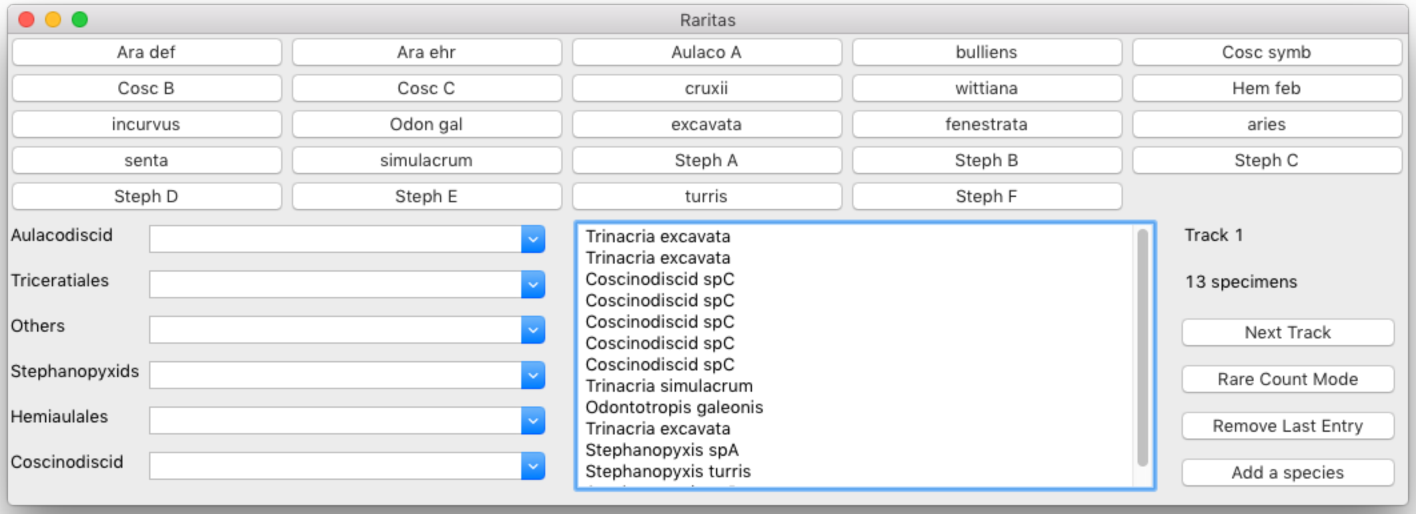

Main window during Normal count mode.

Counting is initially set to 'all taxa' mode, e.g. there is a one to one correspondence to the number of specimens listed in the count history pane and the 'nn specimens' counter. Counting is done by clicking on a taxon button, or by selecting a taxon name from one of the drop-down lists. Each taxon count is listed in the pane in the lower center. The list scrolls as it exceeds the window size. A scroll bar allows you to see the full history of counts however. The specimen counter increments each time a taxon is counted. If you make a mistake you can correct it (if you notice it immediately) with the 'Remove Last Entry' button. You cannot edit the list of older counts however. The entire list can be selected and copied into a separate program (e.g. a text editor) at any time, giving a full history of the observations.

The 'Next Track' button is used to tell the program how much area/effort has been expended generating the counts. If the user does not intend to use 'Rare Count' mode the track button is optional. It is essential to use this if 'Rare Count' mode is used, as the number of tracks counted when entering Rare Count mode is used to estimate the counts of common taxa that are being skipped. The Next Track button should be clicked when a fixed, uniform amount of effort has been done. The term 'track' comes from scanning a single narrow strip of area along a microscrope slide, but can mean any other standard unit of observational effort - fields of view examined, hours spent watching a locality in a visual field survey, etc.

Several options are available while counting (either in 'all taxa', or 'Rare Count' mode as described below) in the View Menu:

Inspect Entries.– This opens a new window with a summary of the observations so far, giving the numbers and relative percent of each taxon counted (counts from the current track are not included). The window is updated once per track, so if you leave it open it will not change to reflect more counts - close the window and reselect the menu option to refresh the values once a new track is started.

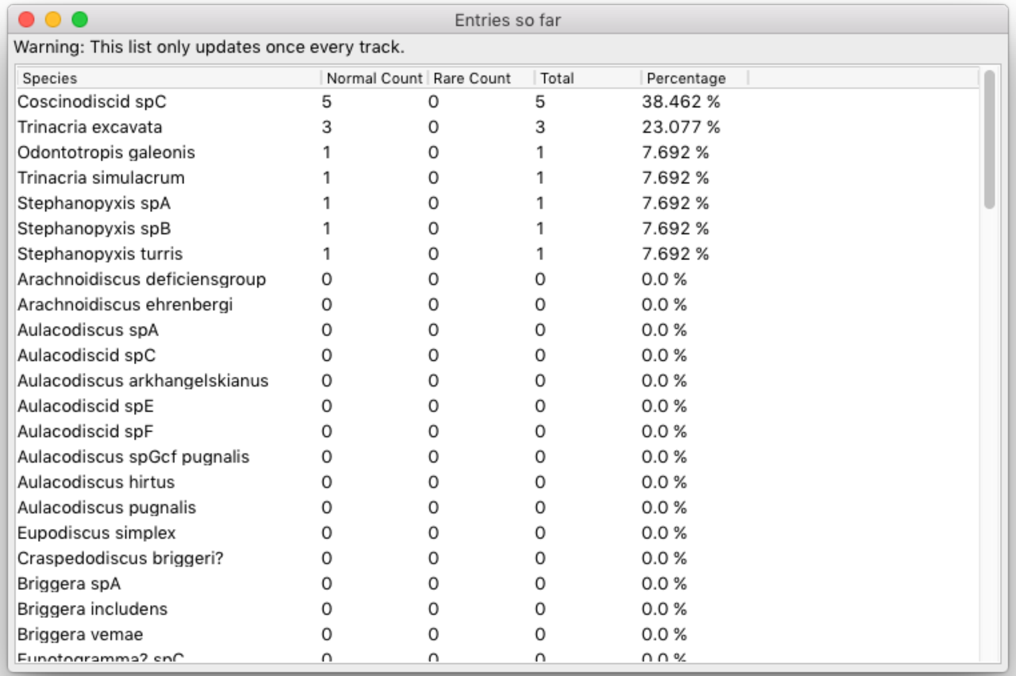

Species	Normal Count	Rare Count	Total	Percentage
Coscinodiscid spC	5	0	5	38.462 %
Trinacria excavata	3	0	3	23.077 %
Odontotropis galeonis	1	0	1	7.692 %
Trinacria simulacrum	1	0	1	7.692 %
Stephanopyxis spA	1	0	1	7.692 %
Stephanopyxis spB	1	0	1	7.692 %
Stephanopyxis turris	1	0	1	7.692 %
Arachnoidiscus deficiensgroup	0	0	0	0.0 %
Arachnoidiscus ehrenbergi	0	0	0	0.0 %
Aulacodiscus spA	0	0	0	0.0 %
Aulacodiscid spC	0	0	0	0.0 %
Aulacodiscus arkhangelskianus	0	0	0	0.0 %
Aulacodiscid spE	0	0	0	0.0 %
Aulacodiscid spF	0	0	0	0.0 %
Aulacodiscus spGcf pugnalis	0	0	0	0.0 %
Aulacodiscus hirtus	0	0	0	0.0 %
Aulacodiscus pugnalis	0	0	0	0.0 %
Eupodiscus simplex	0	0	0	0.0 %
Craspedodiscus briggeri?	0	0	0	0.0 %
Briggera spA	0	0	0	0.0 %
Briggera includens	0	0	0	0.0 %
Briggera vemae	0	0	0	0.0 %
Eunotogramma? spC	0	0	0	0.0 %

Inspect Entries.

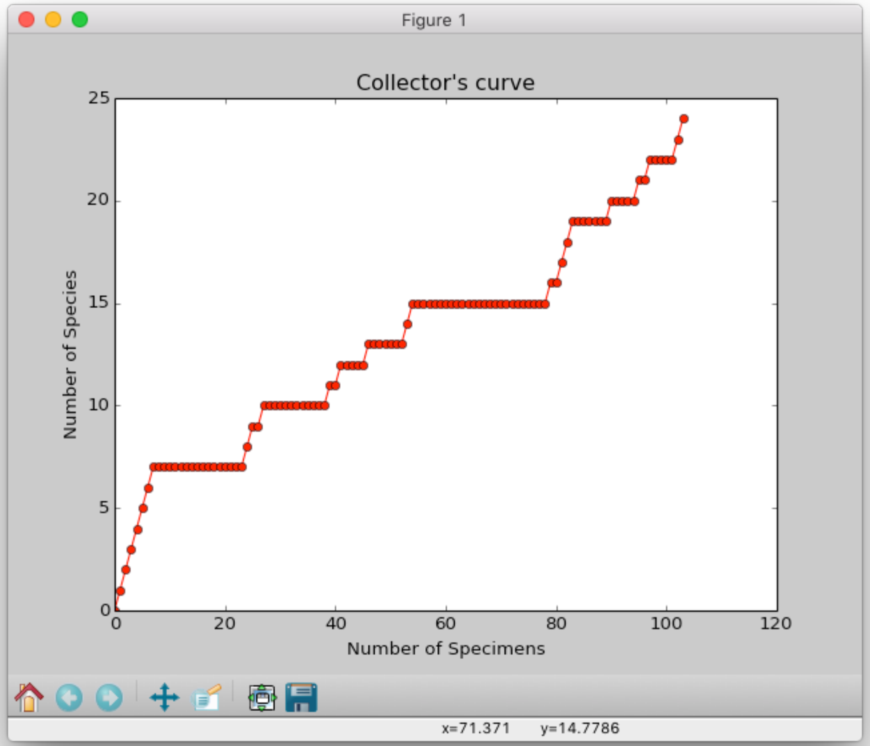

Collector's Curve.

Show Collector's Curve.– A graphic is shown in a separate window - a plot of the number of specimens (on the x-axis) counted vs the cumulative diversity (labeled 'Number of Species') on the y-axis. The window is not dynamically updated and needs to be closed and re-opened to get a current view. The curve provides the user feed-back on how complete the diversity sampling effort is. Such curves usually rise rapidly at first but then flatten out, eventually becoming completely flat as all taxa in the sample have been encountered at least once. See the main publication text and citations given there for more details on how to use this to optimize count effort. The graphic can be saved in one of several graphic formats (.png, .pdf, etc) by clicking the small floppy disc icon in the lower left of the plot window. The other icons do not work reliably (they are generated automatically by the underlying python graphics library that makes the plot) and it is recommended not to use them.

Save Diversity data.– This View menu command saves a simple file containing 2 columns - specimens and species. This gives the data shown in the Collector's Curve graph, and can be used in independent data plots.

Add New Species.– If you encounter taxa while counting that were not provided for in the list created from the configuration file you can add these 'on-the-fly' while counting. You cannot create a new button once the program has started but you can add the species to one of the drop-down lists. Click the 'Add a species' button on the right-hand side of the main counting window. A dialog appears asking for the taxon name and other information (same fields as in the configuration file, with the same constraints on content). When you click 'ok' the species will be added to the end of the drop-down list you chose. This does not currently automatically update the configuration file: beware of this fact when loading an unfinished count Note that creating the species does not automatically add a new count. You need to select it in the list to count it.

3.2 Rare Count Mode

This feature of Raritas allows you to separate your counting effort into two phases - an all taxa phase, and a rare count phase, which allows you to more efficiently count the rarer taxa by ignoring the common ones. See the publication text for more conceptual details. Here only the program functions that support this are described.

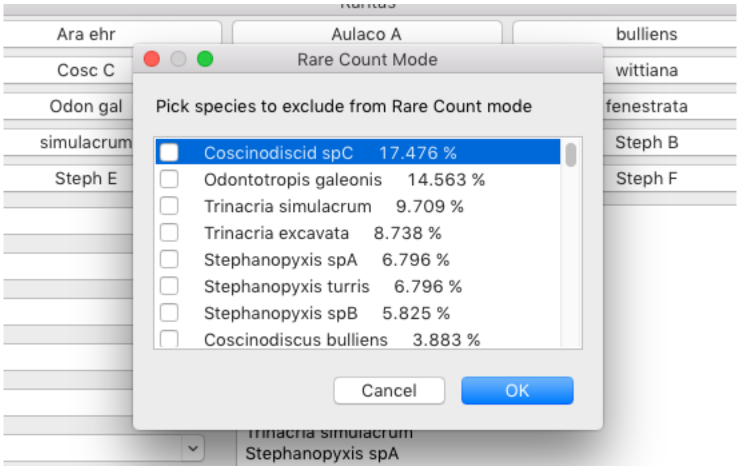

Picking up species to exclude.

The first phase - the 'all taxa' mode described above, gives you the opportunity to count enough specimens to get adequately large numbers of counts for the more common taxa. Once this is done, click on the 'Rare Count Mode' button on the right side of the main counting window. A dialog appears giving a list of taxa counted, sorted by their relative abundance from most to least abundant. A check box is shown in front of each taxa name. Check those species which you feel you have adequate data for (normally the few most abundant taxa, which often make up the majority of the specimens encountered). Click 'ok'. The dialog dissapears and you are returned to the main counting window. You are now in Rare Count mode (as visually identifiable by the greyed out Rare Count Mode button), and the taxa you selected in the dialog will now no longer be counted. You should ignore any specimens that you see of these taxa and continue to scan and count only the remaining (i.e. rarer) taxa. Buttons for these 'taxa to be skipped' will be greyed-out and cannot be clicked anymore. The taxa will still appear in the drop-down lists but if you select one of these taxa it will not be added to the counts. Instead a warning message will appear in

the running list of counts pane - '!Species estimated in rare count mode'. You cannot toggle between modes - once you have entered Rare Count mode you cannot return to 'all taxa' mode.

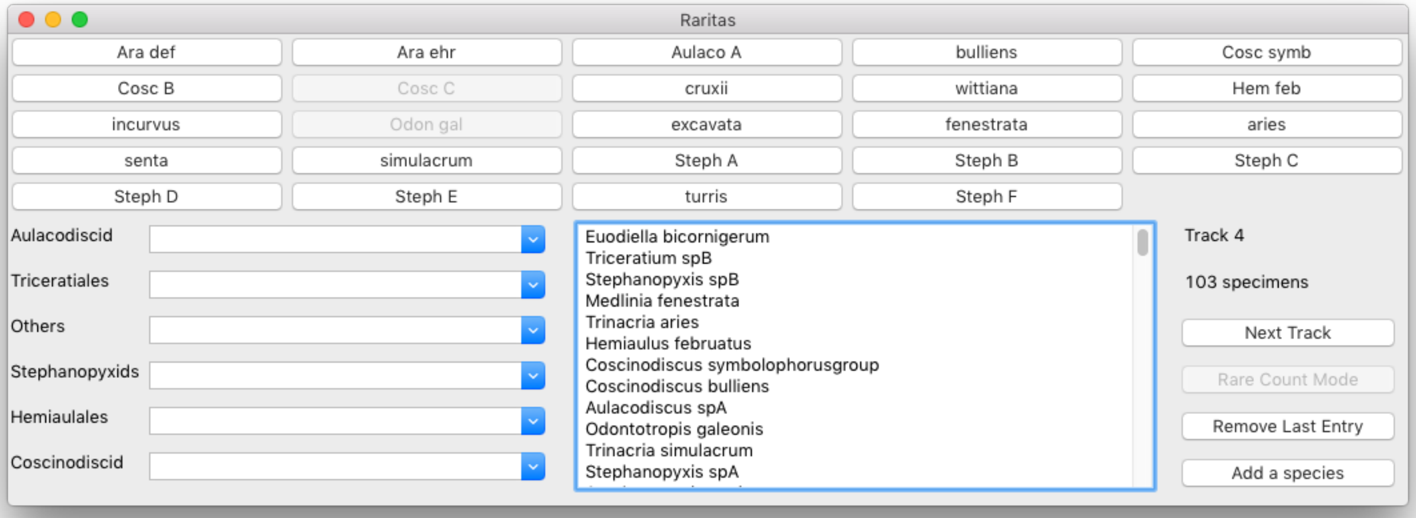

Main Window during Rare count mode.

Certain display functions behave a bit differently in Rare Count mode. The specimen counter will initially increment by one for each new specimen counted, but when the 'Next Track' button is clicked, the numbers of common specimens that were skipped over will be estimated automatically, based on how many of these were seen per track in 'all taxa' mode, and these estimated additional individuals will be added as a block to the 'n specimens' counter. The 'Collector's Curve' graphic will also display these added individuals as blocks, creating noticable steps in the curve. The 'Inspect entries' menu function will show the number actually counted for species in 'normal' mode and in rare count mode, plus, only for those taxa being skipped in Rare Count mode, total counts that, in the rare count column, include the estimated numbers of specimens skipped. These can be identified as they are given as real numbers (with a decimal point) rather than integers.

Note that when you save counts that have been generated in part using Rare Count mode, the counts saved are the actual counted specimens, *Plus*, though only for those common taxa that were skipped in Rare Count mode, the estimated numbers of skipped over specimens calculated by Raritas.

3.3 Saving the Data

Saving the finished set of counted data

Select 'Save finished count' from the programs File menu. You will be asked for a file name and location. The file is saved in SOD-OFF format (described in detail in the publication) - a tab-text tabel with the general metadata entered in the first dialog when Raritas was started in the upper left, the sample specific data as a multirow column header block above the data on the right, and the taxa name information from the configuration file on the left. Counts of common taxa that were made by a combination of actual counts and estimates in Rare Count mode will be identified by 'Estimated based on xx tracks' (where xx is the number of tracks counted before Rare Count mode was activated) in the Taxon Comments field of the output file.

In addition to the main SOD-OFF file of counts for each taxon, a separate file containing 2 columns - specimens and species, is automatically generated using the same file name as the main file with the prefix '`Div_`'. This is the same file that is generated by the 'Save Diversity data' View menu command.

Saving an incomplete set of counts so that the work can be interrupted and resumed later

This brings up a standard file dialog. Name the file and click 'Save'. This file has as columns the cumulative diversity, the list of specimens counted, and the count mode (normal, e.g. all taxa; or rare), plus, if rare count mode was active, the names of the taxa that are being skipped in rare count mode. The information in this file can in

principle be used to calculate diversity and relative abundances independently of the Raritas program. To resume counting, start the program, enter the initial metadata values if needed, then immediately select 'Load unfinished count' from the file menu of the program. The counts and program mode will be restored to where you were when the file was saved.

3.4 Ending the Program

End the program by selecting quit in the menu, or quitting the app using the operating system options, e.g. from the icon in the Mac OS sidebar. Due to a peculiarity of the python modules used to create Raritas, sometimes quitting only closes a single window and the quit command must be selected a second time to fully end the program. Warning! Raritas does NOT check to see if you have saved your count data! If you select 'quit' the program simply ends. Please be sure you have saved your data first!

4 Troubleshooting

Program will not start:

First, make sure you are using a version of of the operating system that is compatible with the app. Raritas for Mac has been tested on OS X versions 10.7 (Lion) through 10.12 (Sierra), and Windows 7 - 10, but is not known to be backwards-compatible with older versions of either OS.

Is the configuration file correctly formatted; are all taxa names unique; is there at least one taxon marked with a 'y' for presentation as a button?

Is the program still running? - As noted above, sometimes parts of the program will still be running even after a quit command has been given. Similarly, start the app from its icon if possible.

Odd behavior when running:

One or more drop-down lists are not visible - drag the main counting window to make it bigger. With numerous lists some will be cut-off at first until the window is made larger.

Do the counts shown on-screen differ from what you expect? You may be in Rare Count mode and some of the counts are estimates of skipped taxa - see the description of this above in Rare Count mode.

Window is too large, or top of window is off-screen. This can happen, particularly if the number of buttons is too large to fit available space. Reduce the number of taxa to be shown as buttons by changing the configuration file and re-run the program. Note for Mac users - you can move windows even if the title bar is off-screen. The method varies by version of the Mac operating system, but in recent OS versions try dragging a vertical edge of a window downwards while pressing the command key.
